# Metagenomic Mining for Esterases in the Microbial Community of Los Rueldos Acid Mine Drainage Formation

**DOI:** 10.3389/fmicb.2022.868839

**Published:** 2022-05-19

**Authors:** Paula Vidal, Mónica Martínez-Martínez, Laura Fernandez-Lopez, Sergi Roda, Celia Méndez-García, Olga V. Golyshina, Víctor Guallar, Ana I. Peláez, Manuel Ferrer

**Affiliations:** ^1^Institute of Catalysis, Department of Applied Biocatalysis, Consejo Superior de Investigaciones Científicas, Madrid, Spain; ^2^Department of Life Sciences, Barcelona Supercomputing Center, Barcelona, Spain; ^3^Área de Microbiología, Departamento Biología Funcional e Instituto de Biotecnología de Asturias, Universidad de Oviedo, Oviedo, Spain; ^4^Centre for Environmental Biotechnology, School of Natural Sciences, Bangor University, Bangor, United Kingdom; ^5^Institució Catalana de Recerca i Estudis Avançats, Barcelona, Spain

**Keywords:** acidophiles, acidophilic bacteria, acid mine drainage, biodiversity, extremozymes, esterase, metagenomics, plastic

## Abstract

Acid mine drainage (AMD) systems are extremely acidic and are metal-rich formations inhabited by relatively low-complexity communities of acidophiles whose enzymes remain mostly uncharacterized. Indeed, enzymes from only a few AMD sites have been studied. The low number of available cultured representatives and genome sequences of acidophiles inhabiting AMDs makes it difficult to assess the potential of these environments for enzyme bioprospecting. In this study, using naïve and *in silico* metagenomic approaches, we retrieved 16 esterases from the α/β-hydrolase fold superfamily with the closest match from uncultured acidophilic *Acidobacteria, Actinobacteria (Acidithrix, Acidimicrobium*, and *Ferrimicrobium), Acidiphilium*, and other *Proteobacteria* inhabiting the Los Rueldos site, which is a unique AMD formation in northwestern Spain with a pH of ∼2. Within this set, only two polypeptides showed high homology (99.4%), while for the rest, the pairwise identities ranged between 4 and 44.9%, suggesting that the diversity of active polypeptides was dominated not by a particular type of protein or highly similar clusters of proteins, but by diverse non-redundant sequences. The enzymes exhibited amino acid sequence identities ranging from 39 to 99% relative to homologous proteins in public databases, including those from other AMDs, thus indicating the potential novelty of proteins associated with a specialized acidophilic community. Ten of the 16 hydrolases were successfully expressed in *Escherichia coli*. The pH for optimal activity ranged from 7.0 to 9.0, with the enzymes retaining 33–68% of their activities at pH 5.5, which was consistent with the relative frequencies of acid residues (from 54 to 67%). The enzymes were the most active at 30–65°C, retaining 20–61% of their activity under the thermal conditions characterizing Los Rueldos (13.8 ± 0.6°C). The analysis of the substrate specificity revealed the capacity of six hydrolases to efficiently degrade (up to 1,652 ± 75 U/g at pH 8.0 and 30°C) acrylic- and terephthalic-like [including bis(2-hydroxyethyl)-terephthalate, BHET] esters, and these enzymes could potentially be of use for developing plastic degradation strategies yet to be explored. Our assessment uncovers the novelty and potential biotechnological interest of enzymes present in the microbial populations that inhibit the Los Rueldos AMD system.

## Introduction

In biotechnology, there is high interest in finding enzymes with new or improved properties (Pellis et al., 2017; Ferrer et al., 2019). This interest is especially increased in relation to enzymes from extremophiles, which are microorganisms that have evolved to thrive in extreme environments (Baweja et al., 2016), as they can efficiently operate under multiple conditions requested by industry. One example application is the eco-friendly bioconversion of cellulosic biomass by extremozymes, which produces green products and has less substrate loss (Thapa et al., 2020). Furthermore, plastic disposal is one of the major problems currently faced by the environment, as enormous quantities of synthetic plastics are non-degradable. Researchers are constantly exploring new ways to degrade plastics, and one of these ways involves using enzymes from microorganisms or microbial communities, including some that inhabit extreme environments (Nchedo Ariole and George-West, 2020).

Acid mine drainage (AMD) systems deserve special attention as a source of extremozymes. AMDs are extremely acidic runoff formations that originate from the microbial oxidation of pyrite and other sulfide minerals, which results in the production of sulfuric acid and metal-rich solutions (Méndez-García et al., 2015; Johnson and Quatrini, 2020). AMD systems are common in our planet, although only a limited number of them have been microbiologically characterized (Méndez-García et al., 2015; Johnson and Quatrini, 2020). Although it has recently been demonstrated that some of these AMD formations, such as the Los Rueldos mercury mine in northwestern Spain (Méndez-García et al., 2014), appear to be populated by a great diversity of prokaryotes, the majority of them are inhabited by a restricted set of acidophilic bacteria and archaea (Dopson et al., 2004; Golyshina, 2011; Méndez-García et al., 2015; Chen et al., 2016; Johnson and Quatrini, 2020), whose variety and abundance depend on geochemical constraints (Méndez-García et al., 2014, 2015; Huang et al., 2016).

Major bacterial lineages detected in AMD systems include the phyla *Proteobacteria* (*Acidithiobacillus, Acidiphilium*, *Acidocella*, *Acidicaldus*, *Acidomonas*, *Acidisphaera*, “*Ferrovum*,” *Acidibacter*, and *Metallibacterium* spp.), *Nitrospirae* (*Leptospirillum* spp. such as *Leptospirillum ferrooxidans*, *Leptospirillum ferriphilum*, and “*Leptospirillum ferrodiazotrophum*”), *Actinobacteria*, *Firmicutes* (*Sulfobacillus* spp., and *Alicyclobacillus* spp.), *Acidobacteria*, *Saccharibacteria* (TM7) and other candidate phyla radiation (CPR) organisms. Archaea include the phyla *Euryarchaeota* (*Ferroplasma* spp. such as *Ferroplasma acidiphilum* and “*Ferroplasma acidarmanus*,” *Acidiplasma cupricumulans*, and *Cuniculiplasma divulgatum*), *Thaumarchaeota*, and the Candidate divisions “*Micrarchaeota*” and “*Parvarchaeota*” (Golyshina et al., 2000, 2009, 2016; Dopson et al., 2004; Baker et al., 2006, 2010; Golyshina, 2011; Chen et al., 2016, 2018; Gavrilov et al., 2019; Korzhenkov et al., 2019). These microorganisms are expected to be reservoirs of enzymes selected to resist acidic harsh conditions (at least regarding extracellular products) (Sharma et al., 2012), some of which might be of biotechnological relevance (Gomes et al., 2003; Adrio and Demain, 2014).

In this category, esterases and lipases from the α/β-hydrolase fold superfamily are appropriate biocatalysts for use in a modern circular bioeconomy because of their abundance (at least one per genome; Ferrer et al., 2015); the extensive knowledge that has been accumulated after the analysis of the biochemical features, sequences, and structures of more than 280,638 such proteins (Bauer et al., 2020); their ease of identification (multiple available screening methods; Reyes-Duarte et al., 2012); and their outstanding properties in terms of stability, reactivity, and scalability, which make them third-choice tools for the functionalization and modification of low-reactivity hydrocarbon-like blocks, oils, and fats (Daiha et al., 2015). Genomics and metagenomics can potentially make accessible an enormous reserve of such important biocatalysts in organisms or microbial communities inhabiting any environment, including AMD systems. However, only 239 of the 280,638 sequences available at the Lipase Engineering (LED) Database (Bauer et al., 2020) have been retrieved from cultured microorganisms (listed above) and uncultured microorganisms that are inhabitants of AMD systems, including *Alicyclobacillus* spp., 118 in total; *Sulfobacillus* spp., 53; *Acidobacteria*, 34; *Acidithiobacillus*, 13; *Leptospirillum*, 9; “*Ferrovum*,” 5; *Acidocella*, 3; and *Ferroplasma*, *Aciditrix*, *Acidiphilium*, and *Metallibacterium*, with 1 each. Among these biocatalysts, only a low-pH optimum carboxylesterase from *F. acidiphilum* (Ohara et al., 2014) has been characterized. This limits the assessment of the biotechnological potential of acidophiles living in AMD systems, at least regarding esterases and lipases. The minimal enzyme-level information that is known about these systems is restricted to two endo-acting amylases with no similarity to any known protein and two genes conferring metal and acid resistance from the microbial community inhabiting the AMD systems of the Carnoulès (lead–zinc) mine in France (Delavat et al., 2012) and the Tinto River in southwestern Spain (Delavat et al., 2012; Guazzaroni et al., 2013), respectively.

To fill this knowledge gap, we initiated a metagenomic investigation to isolate carboxylesterases from a recently discovered and microbiologically characterized AMD formation, namely, the Los Rueldos mercury mine in northwestern Spain (Méndez-García et al., 2014). By applying homology searches in metagenomic sequences and naïve screening in clone libraries with enzyme substrates, we discovered a number of such enzymes whose characteristics are reported herein. Both function- and DNA sequence-based metagenomic methods are complementary, with each having advantages and disadvantages. Bioinformatics methods allow a rapid process of enzyme searching. However, in prokaryotic genomes, >30% of genes remain annotated as “hypothetical, conserved hypothetical or with general prediction,” and large numbers of genes may have non-specific annotations (such as putative hydrolases). The analysis of biochemical functions is likely to provide a superior approach to avoid this limitation, especially when screening novel enzymes. However, only a few hundred specific enzymatic assays exist, with a limited number of them applied in a high-throughput manner for the naïve screening of metagenomics libraries.

Although the *in vivo* roles and expression levels of the genes encoding the hydrolases presented in this study are unknown, their sequences and results of biochemical analyses shed new light on the enzymology of the microbial inhabitants of the Los Rueldos AMD formation, which have been neglected in enzyme prospecting.

## Materials and Methods

### General Experimental Procedures

The source and brand of each of the esters (purity ≥99%) used in this study was Merck Life Science S.L.U., Madrid, Spain. The oligonucleotides used for DNA amplification were synthesized by Sigma Genosys, Ltd. (Pampisford, Cambridgeshire, United Kingdom). The *Escherichia coli* EPI-300-T1R strain used for pCCFOS1 fosmid library construction and screening was from Epicentre Biotechnologies (Madison, WI, United States). The *E. coli* strain GigaSingles used for gene cloning and *E. coli* strain BL21 (DE3) used for gene expression were from Novagen (Darmstadt, Germany).

### Sampling Site and Sample Collection

The Los Rueldos gallery is located along the northwestern slope of the Morgao Valley (2 km northeast of the town of Mieres and 20 km southeast of Oviedo, which is the capital city of Asturias in northwestern Spain; 43°15′47″N, 5°46′9″W). It is a 70 m-long gallery with 10–30 cm depths in the shallower areas and 40–70 cm depths in the deeper sections (Méndez-García et al., 2014). Microorganisms are developed along the AMD system (pH ∼2), forming a bedded acidic biofilm with uppermost oxic (B1A) and lowermost anoxic (B1B) strata. The DNA samples from B1A and B1B (see below) samples collected and used in this study were the same as those in the previous work (Méndez-García et al., 2014). Briefly, samples were collected in sterile 50 ml tubes at two sampling sites determined by the presence of each different macroscopic microbial growth morphology (B1A: up to 2 cm deep; B1B: from 2 to 15 cm deep) and kept on ice until nucleic acid extraction was performed (within the following 2 h).

### Nucleic Acid Extraction, Preparation of pCCFOS1 Libraries, and Naïve Screening

The DNA samples from B1A and B1B were the same as those used in a previous work (Méndez-García et al., 2014), which were obtained using the Power Soil DNA extraction kit (Cambio, Cambridge, United Kingdom) according to the manufacturer’s guidelines. Prior to clone library construction, the metagenomics DNA was concentrated by first adding 50 μl of 3 M sodium acetate solution to 50 μl DNA extract. Precipitation was conducted by the addition of 1.25 ml of ethanol and incubation at room temperature for 10 s. Precipitated DNA was pelleted by centrifugation at 20,000 *g* for 10 min. The resulting pellets were washed with 500 μl of 70% (v/v) ethanol twice, and the traces of ethanol were evaporated by incubation under a fume hood at room temperature for 10 min. The resulting pellets were then dissolved in 20 μl of sterile nuclease-free water. Before cloning in the large-insert pCCFOS1 fosmid libraries using the CopyControl Fosmid Library Kit (Epicentre Biotechnologies, Madison, WI, United States) and the *E. coli* EPI300-T1*^R^* strain, the DNA (10 μg) that was unsheared by gel electrophoresis was subjected to shearing by pipetting through a 200 μl pipette tip 100 times, following the recommendations of the supplier (Epicentre Biotechnologies, Madison, WI, United States) to reach an approximately size of 30,000 bp. Cells of each pCCFOS1 fosmid library were suspended in glycerol to a final concentration of 20% (v/v) and stored at −80°C until further use. We generated subsets of 94,000 and 81,000 clones for the B1A and B1B samples, respectively. Restriction analysis of 10 randomly selected clones from each library revealed average insert sizes of 34,000 bp (for the B1A samples) and 39,500 bp (for the B1B samples), which included nearly 3.2 Gbp of community genomes per sample. This size is within the range of the average size range of DNA inserts in positive clones found in this study (see below).

Fosmid clones were plated onto large (22.5 × 22.5 cm) Petri plates with Luria Bertani (LB) agar containing chloramphenicol (12.5 μg/ml) and induction solution (Epicentre Biotechnologies; WI, United States) at a quantity recommended by the supplier to induce a high fosmid copy number. Clones were scored by the ability to hydrolyze α-naphthyl acetate (α-NA) and tributyrin, as previously described (Reyes-Duarte et al., 2012). Positive clones presumptively containing carboxylesterases and lipases with the α/β hydrolase fold were selected, and their DNA inserts were sequenced using a MiSeq Sequencing System (Illumina, San Diego, CA, United States) with a 2 × 150-bp sequencing v2 kit at Lifesequencing S.L. (Valencia, Spain). Before sequencing, fosmid DNA was extracted from the fosmid clones containing the metagenomic segments using the QUIAGEN Large-Construct Kit (QUIAGEN, Hilden, Germany), according to the manufacturer’s protocol. Upon the completion of sequencing, the reads were quality-filtered and assembled to generate non-redundant meta-sequences, and genes were predicted and annotated as described previously (Placido et al., 2015).

### Selection of Genes Encoding Enzymes by Homology Sequence Analysis

The predicted protein-coding genes obtained in a previous study (Méndez-García et al., 2014) after the sequencing of DNA material from resident microbial communities in each of the samples (B1A and B1B) with a Roche 454 GS FLX Ti sequencer (Roche Applied Science, Penzberg, Germany) were used in this study. The meta-sequences are available from the National Center for Biotechnology Information (NCBI) non-redundant public database with the IDs PRJNA193663 (for B1A) and PRJNA193664 (for B1B). Protein-coding genes identified from metagenomes (sequence-based screening) and from the DNA inserts of positive clones (naïve screen) were screened (score >45; e-value <10e^–3^) using BLASTP and PSI-BLAST searching (Altschul et al., 1997) for enzymes of interest against the *ESTerases and alpha/beta-Hydrolase Enzymes and Relatives* (ESTHER) and LED databases (Fischer and Pleiss, 2003; Barth et al., 2004; Bauer et al., 2020).

### Gene Expression and Protein Purification

The experimental procedures used for the cloning, expression, and purification of selected proteins (either from naïve or homology sequence screening) in the Ek/LIC 46 vector and *E. coli* strain BL21 (DE3) were performed as described previously (Alcaide et al., 2015). The primers used for amplification are listed in Supplementary Material. All proteins studied here were N-terminally His_6_-tagged, and the soluble His-tagged proteins were produced and purified at room temperature after binding to a nickel–nitrilotriacetic acid (Ni–NTA) His-Bind resin (from Merck Life Science S.L.U., Madrid, Spain) as described previously (Giunta et al., 2020), with slight modifications (the expression culture was scaled up to 1 L using 50 ml pre-inoculum). The purity was assessed as >98% using sodium dodecyl sulfate-polyacrylamide gel electrophoresis (SDS–PAGE; Supplementary Figure 1) in a Bio-Rad Mini Protein system (Laemmli, 1970). Protein concentrations were determined according to the Bradford method with bovine serum albumin as the standard (Bradford, 1976). A total of approximately 0.8–37 mg of purified recombinant proteins was obtained from each 1 L culture on average, as follows: Est_*A*1_ (6.4 mg/L), Est_*A*2_ (25 mg/L), Est_*A*3_ (13 mg/L), Est_*A*4_ (37 mg/L), Est_*A*5_ (41 mg/L), Est_*A*6_ (7 mg/L), Est_*A*7_ (0.8 mg/L), Est_*A*8_ (19 mg/L), Est_*B*1_ (1.0 mg/L), and Est_*B*2_ (32 mg/L).

### Enzyme Assays

The hydrolysis of 2-naphthyl acrylate (ref. 577189), tri(propylene glycol) diacrylate (ref. 246832), dibenzyl terephthalate (ref. PH000126), and bis(2-hydroxyethyl)-terephthalate (BHET; ref. 465151) (all from Merck Life Science S.L.U., Madrid, Spain) was assessed using a pH indicator assay in 384-well plates (ref. 781162, Greiner Bio-One GmbH, Kremsmünster, Austria) at 30°C and pH 8.0 in a Synergy HT Multi-Mode Microplate Reader in continuous mode at 550 nm over 24 h [extinction coefficient (ε) of phenol red, 8,450 M^–1^ cm^–1^]. The acid produced after ester bond cleavage by the hydrolytic enzyme induced a color change in the pH indicator that was measured spectrophotometrically at 550 nm. The experimental conditions were as detailed previously (Giunta et al., 2020), with the absence of activity defined as at least a twofold background signal. For *V*_*max*_ determination, (protein): 270 μg/ml; (ester): 20 mM; reaction volume: 44 μl; T: 30°C; and pH: 8.0. Activity was calculated by determining the absorbance per minute from the generated slopes and applying the following equation:


$$Rate\left(\frac{\mu mol}{\min mgprotein}\right)=\frac{\frac{△\text{Abs}}{\text{min}}}{8450M^{-1}{\text{cm}}^{-1}}*\frac{1}{0.4cm}*\frac{10^{6}\mu M}{1M}*0.000044L*\frac{1}{mgprot.}$$

The activity toward the model esters *p*-nitrophenyl acetate (*p*NPC_2_), propionate (*p*NPC_3_), butyrate (*p*NPC_4_), octanoate (*p*NPC_8_), decanoate (*p*NPC_10_), and decanoate (*p*NPC_12_) was assessed in 50 mM Britton and Robinson (BR) buffer at pH 8.0 and 30°C by monitoring the production of 4-nitrophenol at 348 nm (pH-independent isosbestic point, ε = 4147 M^–1^ cm^–1^) for over 5 min and determining the absorbance per minute from the generated slopes (Santiago et al., 2018). The reactions were performed at 30°C in 96-well plates (ref. 655801, Greiner Bio-One GmbH, Kremsmünster, Austria) and contained 0.09 to 3 μg proteins and 0.8 mM esters in a total volume of 200 μl. The effect of pH on the activity was determined in 50 mM BR buffer at pH 4.0–12.0, as described previously. Similar assay conditions were used to assay the effects of temperature on the ester hydrolysis of *p*NPC_3_, but in this case, the reactions were performed in 50 mM BR buffer pH 7.0. Note that the BR buffer consists of a mixture of 0.04 M H_3_BO_3_, 0.04 M H_3_PO_4_, and 0.04 M CH_3_COOH that has been titrated to the desired pH with 0.2 M NaOH. All values were determined in triplicate and were corrected for non-enzymatic transformation. In all cases, the activity was calculated by determining the absorbance per minute from the generated slopes and applying the following equation:


$$Rate\left(\frac{\mu mol}{\min mgprotein}\right)=\frac{\frac{△\text{Abs}}{\text{min}}}{4147M^{-1}{\text{cm}}^{-1}}*\frac{1}{0.4cm}*\frac{10^{6}\mu M}{1M}*0.0002L*\frac{1}{mgprotein}$$

Poly(propylene glycol) diacrylate (ref. 455024, Merck Life Science S.L.U., Madrid, Spain) and poly(DL-lactide) with an average molecular weight 2,000 (ref. AP224, PolySciTech, Akina, IN, United States) were assayed as described previously (Hajighasemi et al., 2018). The hydrolysis of polyethylene terephthalate (PET) films (prepared as reported by Bollinger et al., 2020) and particles, which were prepared using PET from a bottle (from a local shop – Granini brand), as described previously (Pütz, 2006), was evaluated at 30°C and pH 8.0 with 270 μg protein/ml and 2 mg/ml plastic material, as previously reported (Bollinger et al., 2020).

The effect of the inhibitors mercaptoethanol (ref. M7154) and iodoacetamide (ref. I1149), which were both from Merck Life Science S.L.U., Madrid, Spain, was tested as follows. A mixture containing the purified enzymes (final concentration of 1 mg/ml) in 190 μl of 40 mM 4-(2-hydroxyethyl)-1-piperazineethanesulfonic acid (HEPES) at pH 7.0 and the inhibitors (final concentration, 1–10 mM) was incubated for 5 min to 24 h at 30–45°C. The reaction was initiated by adding *p*NPC_3_ (0.8 mM, final concentration), and the activity was measured for over 5 min as described above and compared to control samples without inhibitors.

### Circular Dichroism to Estimate Thermal Denaturation

Circular dichroism (CD) spectra were acquired between 190 and 270 nm with a Jasco J-720 spectropolarimeter equipped with a Peltier temperature controller in a 0.1-mm cell at 25°C. The spectra were analyzed, and denaturation temperature (T_*d*_) values were determined at 220 nm between 10 and 85°C at a rate of 30°C per hour in 40 mM HEPES buffer at pH 7.0. CD measurements were performed at pH 7.0 and not at the optimal pH (8.5–9.0) to ensure protein stability. A protein concentration of 0.5 mg/ml was used. T_*d*_ (and the standard deviation of the linear fit) was calculated by fitting the ellipticity (mdeg) at 220 nm at each of the different temperatures using a 5-parameter sigmoid fit with SigmaPlot 13.0.

### Codes and Accession Numbers

The sequences were named based on the code “Est,” which refers to Esterase, followed by a letter indicating the origin of the sample, as follows: Est_*A*_, esterase from the uppermost oxic B1A strata; and Est_*B*_, esterase from the lowermost anoxic B1B sediment attached strata. The final number (subscript) is an arbitrary number representing the number of enzymes per site. Sequences encoding enzymes were deposited under the BioProject IDs PRJNA193663 (for B1A) and PRJNA193664 (for B1B) in the NCBI public database, with the accession numbers detailed in Table 1.

**TABLE 1:** General sequence-based characteristics of Los Rueldos esterases.

Name	Accession number	Contig bp [taxonomic origin (phylum, genus)]*^d^*	Identity and best hit*^e^*	*p*I	Putative catalytic triad*^f^*
EstA_1_*^a,b^*	KY010297	41,280 (*Actinomycetota*, *Acidithrix*)	99%; WP_052605564.1	4.62	Ser_146_, Asp_193_, His_270_
EstA_2_*^a,b^*	KY010298	33,407 (*Actinomycetota*, *Acidimicrobium*/*Ferrimicrobium*)	94%; NNN14078.1	5.03	Ser_185_, Asp_316_, His_412_
EstA_3_*^a,b^*	KY010300	32,091 (*Acidobacteria, a.a.*)	58%; WP_041839843	5.44	Ser_83_, Asp_231_, His_234_
EstA_4_*^a,b^*	KY010299	35,459 (*Proteobacteria, Acidiphilium*)	79%; OYV70855.1	5.53	Ser_159_, Asp_254_, His_284_
EstA_5_*^a,b^*	KY019260	26,956 (*Proteobacteria, a.a.*)	62%; ODU57651.1	5.43	Ser_159_, Asp_254_, His_284_
EstA_6_*^a,b^*	KY010302	39,640 (*Proteobacteria, a.a.*)	62%; ODU57651.1	5.32	Ser_159_, Asp_254_, His_284_
EstA_7_*^b,c^*	EQD63018.1	2,621 (*Proteobacteria, a.a.*)	64%; ODU34315	6.29	Ser_123_, Asp_175_, His_207_
EstA_8_*^a,b^*	KY010301	38,626 (*Proteobacteria, a.a.*)	56%; WP_063671588.1	10.04	Ser_90_, Asp_237_, His_240_
EstA_9_*^c^*	EQD66234.1	1,763 (*Proteobacteria, a.a.*)	53%; WP_055246968.1	7.12	Ser_120_, Asp_188_, His_220_
EstA_10_*^a^*	KY010303	16,545 (*Actinomycetota*, *Acidithrix*)	100%; WP_052605292.1	5.55	Ser_75_, Lys_75_, Tyr_193_
EstA_11_*^a^*	KY010304	40,600 (*Proteobacteria, a.a.*)	67%; WP_051488053	9.71	Asp_506_, His_578_, His_582_
EstA_12_*^a^*	KY010305	35,290 (*Bacteria, a.a.*)	41%; WP_009508720.1	9.65	Ser_109_, Asp_315_, His_318_
EstB_1_*^b,c^*	EQD71191.1	2,283 (*Proteobacteria, a.a.*)	69%; WP_049623914.1	5.89	Ser_117_, Asp_165_, His_294_
EstB_2_*^b,c^*	EQD52136.1	2,483 (*Proteobacteria, a.a.*)	48%; WP_055799051.1	6.11	Ser_116_, Asp_164_, His_195_
EstB_3_*^c^*	EQD26916.1	13,465 (*Proteobacteria, a.a.*)	49%; WP_026633329.1	5.7	Ser_59_, Asp_313_, His_316_
EstB_4_*^c^*	EQD55146.1	13,877 (*Actinobacteria, a.a.*)	39%; WP_051823767.1	6.07	Ser_86_, Asp_202_, His_231_

*^a^Identified by naïve screen.*

*^b^Protein expressed as active soluble form.*

*^c^Identified by sequence-based screen from metagenomic sequencing data; accession number of the sequences in NCBI being ID PRJNA193663 (for B1A) and PRJNA193664 (for B1B).*

*^d^As determined by GOHTAM database (Ménigaud et al., 2012) and TBLASTX; taxonomic assignation to phylum and genera are shown, with a.a. defining those for which ambiguous assignations below phylum level were obtained.*

*^e^Best hit and identity as shown by TBLASTX.*

*^f^Presumptive catalytic triad as found by 3D structure analysis and alignment with homologous proteins with solved crystal structures.*

### Three-Dimensional Modeling

The models of the protein structures were predicted with AlphaFold 2.1.0 (Jumper et al., 2021).

## Results

### Enzyme Selection and Divergence at the Sequence Level

Microbial communities inhabiting two distinct compartments within Los Rueldos AMD formation were screened for sequences encoding carboxylesterases or lipases. For that, we used two complementary metagenomics approaches, namely, naïve and homology sequence screens. First, a total of approximately 81,000 pCCFOS1 clones from each clone library (equivalent to 2.8 Gbp for B1A and 3.20 Gbp for B1B) were screened for esterase/lipase activity using plate-based screen with αNA and tributyrin as model substrates. We identified a total of 10 positive clones being active against both substrates in the B1A clone library, whereas no positives were found in the B1B library. The fosmids with insert lengths ranging from 16,545 to 41,280 bp were fully sequenced, from which 10 genes (one per positive clone), encoding presumptive esterases/lipases, were identified. In addition, we searched the predicted protein-coding genes obtained through next-generation sequencing for sequences encoding esterases and lipases by BLAST search against the ESTHER and LED databases. A total of 6 full-length sequences (B1A: 2; B1B: 4), with accession numbers EQD63018.1, EQD66234.1, EQD71191.1, EQD52136.1, EQD55146.1, and EQD26916.1, encoding potential enzymes were identified. Taken together, a total of 16 genes encoding hydrolases from the α/β-hydrolase fold superfamily, specifically, 12 from B1A (EstA_1_ to EstA_12_) and 4 from B1B (EstB_1_ to EstB_4_), were identified (Table 1). As determined by Matcher (EMBOSS package), the pairwise amino acid sequence identity for 14 of the 16 α/β hydrolases ranged from 4.0 to 44.9%. This, together with the fact that only 2 out of 16 polypeptides were highly similar (EstA_5_ and EstA_6_ differ in only 2 amino acids: arginine 152 and alanine 179 in Est_*A*5_ are cysteine 152 and threonine 179 in Est_*A*6_), suggests a large divergence at the sequence level within the enzymes examined, and that the diversity of polypeptides was not dominated by a particular type of protein or highly similar clusters of proteins, but rather by diverse non-redundant sequences. Note that only 1 of 10 sequences selected after naïve screens was found in the metagenomic data generated after direct DNA sequencing (EstA_11_, which is 99% identical to GenBank accession no. EQD37671.1 from the Los Rueldos metagenome) (Méndez-García et al., 2014). This demonstrates that both types of screens (naïve and *in silico*) are complementary tools for enzyme discovery. However, deeper metagenomic sequencing could potentially detect all enzymes isolated by naïve screens.

The deduced molecular mass and estimated isoelectric point (*p*I) values ranged from 23.19 to 101.53 kDa and from 4.62 to 10.04, respectively. Putative proteins exhibited a maximum amino acid sequence identity ranging from 39 to 100% to putative esterases/lipases in public databases (Table 1). It is worth mentioning that EstA_3_, EstA_8_, and EstB_3_ are related to presumptive esterase/lipase-like subfamily proteins of the Serine-Glycine-Asparagine-Histidine (SGNH) hydrolases, EstA_11_ to presumptive glycoside-hydrolase family GH114 (N-terminal domain) and CE4_PelA_like hydrolases (C-terminal domain), and EstA_12_ to presumptive sialate O-acetylesterases. A further TBLASTX search against metagenomics proteins deposited in databases revealed no similarity of 10 proteins with homologous AMD metagenome proteins. In contrast, five proteins (EstA_3_ to EstA_6_, and EstA_12_) do share from 27 to 54% homology to three proteins from the Carnoules arsenic-contaminated mine drainage (GenBank: CBI07622.1, CBH97521.1, and CBI00527.1). This finding suggests that esterases/lipases from microbial communities from the Los Rueldos site are distantly related to proteins from other known homologous proteins from AMD formations with metagenome sequences available. It also reflects the large undiscovered pool of enzymes from bacterial species populating the Los Rueldos site.

### Primary Structure Analysis

**FIGURE 1:**
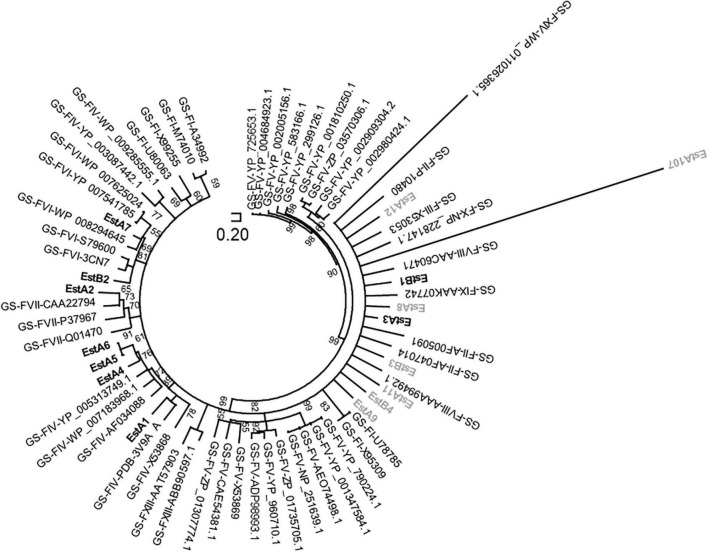
The unrooted circular neighbor-joining tree indicating phylogenetic positions of polypeptide sequences of Los Rueldos esterases. Positioning is referred to homologous proteins with unambiguous categorization into lipase/esterase families (from Family I to XIV) according to Arpigny and Jaeger and further classifications (Arpigny and Jaeger, 1999; Rao et al., 2013). GS-F, genome sequences assigned to an esterase/lipase family (in bold letters). Sequences from Los Rueldos that correspond to proteins that could not be produced as soluble active proteins using Ek/LIC 46 vector and *E. coli* strain BL21 (DE3) as a host are indicated in gray color, while those being active and soluble are indicated in bold.

Based on the comparison of the primary structures, 14 families of sequence-related esterases and lipases have been reported (Arpigny and Jaeger, 1999; Rao et al., 2013). Sequence analysis categorized 13 enzymes from Los Rueldos into some of these known subfamilies (Figure 1) with most structurally similar homologs as follows: EstA_9_ [27%; best hit in Protein Data Bank (PDB) 3DOH_A] and EstB_4_ (41%; 3OM8_A) to Family I; EstA_1_ (41%; 3V9A_A), EstA_4_ (41%; 4YPV_A), EstA_5_ (49%; 4YPV_A), and EstA_6_ (49%; 4YPV_A) to Family IV; EstA_7_ (53%; 4YPV_A) and EstB_2_ (41%; 1AUO_A) to Family VI; EstA_2_ (37%; 2OGT_A) to Family VII (EstA_2_); and EstA_10_ (41%; 4IVK_A) to beta-lactamase like Family VIII. EstA_3_ (43%; PDB code 3P94_A), EstA_8_ (44%; PDB code 3P94_A), and EstB_3_ (26%; PDB code 3KVN_X) belong to Family II GDSL, but the structural alignment also confirms that they contain a domain that displays the characteristic α–β–α globular fold of the SGNH hydrolase family. In addition, EstB_3_ also contains a passenger domain providing the driving force for passenger translocation (Van den Berg, 2010). Three of the sequences could not be assigned to these subfamilies. First, EstA_11_ contains a 300 amino acid long N-terminal domain most similar to glycoside-hydrolase family 114 and a 616 amino acid long C-terminal domain most similar to the carbohydrate esterase 4 (CE4) superfamily that includes chitin deacetylases (EC 3.5.1.41), N-acetylglucosamine deacetylases (EC 3.5.1.-), and acetylxylan esterases (EC 3.1.1.72), which catalyze the N- or O-deacetylation of substrates such as acetylated chitin, peptidoglycan, and acetylated xylan. Its N-terminal domain is most structurally similar (26%) to that of the glycosidase 2AAM_A and its C-terminal domain is structurally similar to the polysaccharide deacetylase from *Bacillus cereus* (4HD5). Second, EstA_12_ is associated with acetylxylan esterases (EC 3.1.1.72), with most similar (21%) structural homolog in PDB being 1ZMB_A. Third, EstB_1_ shows homology to small serine alpha/beta-hydrolase/acyl-peptidase (58%; 2FUK_A). The tentative amino acids participating in the typical catalytic triad of esterases and lipases are summarized in Table 1. Together, the analysis of the primary sequence suggests that the diversity of esterases was not dominated by a particular type of protein or a highly similar cluster of proteins, but rather by diverse non-redundant sequences belonging to different microbial groups and distinct esterase/lipase subfamilies.

### Source Organisms of Selected Polypeptides

A search of oligonucleotide patterns against the GOHTAM database (Ménigaud et al., 2012) and TBLASTX analysis revealed compositional similarities between the DNA fragment containing the genes for EstA_1_, EstA_2_, EstA_10_, and EstB_4_, with genomic sequences of bacteria from the phylum *Actinomycetota*. Among them, only unambiguous affiliations at lower levels could be achieved for fragments containing Est_*A*1_, Est_*A*2_, and EstA_10_ that may most likely belong to bacteria from the genera *Acidithrix* (EstA_1_ and EstA_10_) and *Acidimicrobium*/*Ferrimicrobium* (EstA_2_), both from the family *Acidimicrobiaceae* within the order *Acidimicrobiales*. Note that EstA_1_ and EstA_10_ showed 99–100% sequence identity with uncharacterized esterases and lipases (WP_052605564.1 and WP_052605292.1) from *Acidithrix ferrooxidans*, and EstA_2_ showed 94% sequence identity with an uncharacterized esterase-lipase (NNN14078.1) from *Acidimicrobiaceae*. EstA_3_ was most likely derived from an uncultured bacterium assigned to the phylum *Acidobacteria* with ambiguous affiliation below the phylum level. The genes for EstA_4_ to EstA_9_, EstA_11_, and EstB_1_ to EstB_3_ were associated with uncultured bacteria of the *Proteobacteria* phylum, with ambiguous affiliations at a lower taxonomic level, except for EstA_4_, which was most likely derived from a bacterium of the genus *Acidiphilium* from the family *Acetobacteraceae* within the order *Rhodospirillales* (best hit OYV70855.1 from *Acidiphilium* sp., 79% homology). All these bacterial groups have been detected in biofilms thriving in the Los Rueldos mine (Méndez-García et al., 2014). No clear affiliation, other than Bacteria, could be found for EstA_12_. Note that in some cases no clear affiliation to a taxon of source organism below the level of the phylum could be established, either because of the short fragment length or the low compositional similarity between the metagenomic fragments and the sequences of related bacterial chromosomes and plasmids do not allow proper assignations.

### Enzyme Characterization

From the 16 sequences selected, 8 from B1A and 2 from B1B were successfully cloned, expressed, and purified as soluble active proteins when expressed in pET Ek/LIC 46 vector and *E. coli* BL21 as the host. These proteins were herein referred to as EstA_1_ to EstA_8_, EstB_1_, and EstB_2_. The remaining six (EstA_9_-EstA_12_ and EstB_3_-EstB_4_) could not be produced in soluble active form (they formed inclusion bodies) in the expression system applied herein, which consists of the use of the Isopropyl β-D-1-thiogalactopyranoside (IPTG)-inducible Ek/LIC 46 vector and *E. coli* strain BL21 (DE3) as a host, and their properties are not described herein. Refining the expression conditions, which included variations in the expression conditions (16, 30, and 37°C) and IPTG concentration (from 0.1 to 2 mM), resulted in unsuccessful production of sufficient active protein material for characterization. Further efforts may be needed with different expression vectors, which is beyond the scope of the present study.

The substrate profile of all α/β hydrolases was first examined using a set of esters commonly used to characterize esterases and lipases, namely, *p*NP esters such as *p*NPC_2_, *p*NPC_3_, *p*NPC_4_, *p*NPC_8_, *p*NPC_10_, and *p*NPC_12_. All ester hydrolases preferred short-chain-length pNP-esters, particularly *p*NPC_2_ (EstA_3_), *p*NPC_3_ (EstA_2_, EstA_4_, EstA_5_, EstA_6_, EstB_1_, and EstB_2_), and *p*NPC_4_ (EstA_1_, EstA_7_, and EstA_8_) (Table 2). Within all six *p*NP ester tested, all but one (EstA_3_) enzyme was able to hydrolyze up to *p*NPC_12_, albeit at a much lower level (from 62- to 3,900-fold) compared to shorter derivatives. Considering the preferred *p*NP esters, the maximum specific activity ranged from 3.06 ± 0.03 to 679.8 ± 9.8 U/mg. We further test the possibility that the enzymes hydrolyze substrates other than *p*NP esters, particularly, plastic substrates and esters relevant to plastics. Using previously described conditions (Hajighasemi et al., 2018; Bollinger et al., 2020), we did not find that any of the enzymes hydrolyzed large plastic materials such as poly(propylene glycol) diacrylate, poly(DL-lactide), amorphous PET film, and PET nanoparticles. However, by using a pH-indicator assay, we found that the enzymes were able to hydrolyze other terephthalate esters and acrylate esters. Thus, as shown in Table 3, six of the enzymes hydrolyzed esters relevant to acrylic acid plastics, e.g., 2-naphthyl acrylate and tripropylene glycol diacrylate, a commonly used material principally exploited to prepare thermally stable polymers (He et al., 2017). These substrates, herein found to be converted at a maximum rate of 3,915 ± 48 U/g, are rarely hydrolyzed by esterases and lipases. There are only two examples reported, namely, human salivary pseudocholinesterase and cholesterol esterase (Finer and Santerre, 2004; Cai et al., 2014). In addition, one of the enzymes (Est_*A*8_) was capable of hydrolyzing dibenzyl terephthalate (432.2 ± 27.5 U/g), an intermediate produced during chemical PET recycling with benzyl alcohol in the presence of a catalyst (Donahue et al., 2003). No esterase or lipase has been reported to date that degrades this substrate. In addition, six of the esterases (Est_*A*1_, Est_*A*2_, Est_*A*5_, Est_*A*6_, Est_*A*8_, and Est_*B*2_) efficiently hydrolyzed BHET (from 5.0 ± 1.0 to 336.9 ± 3.6 U/g), an intermediate in the degradation of PET (Yoshida et al., 2016). High performance liquid chromatography (HPLC) analysis, performed as described (Bollinger et al., 2020), confirmed the hydrolysis of BHET to mono-(2-hydroxyethyl)-terephthalic acid (MHET) and not to terephthalic acid. To conclude, the enzymes reported herein from the Los Rueldos AMD formation showed high activity for converting and recycling components of synthetic plastics, namely, acrylic- and terephthalate-based plastics, and could be of potential use in developing plastic degradation strategies.

**TABLE 2:** Specific activity (U/mg pure protein) for each of the enzymes tested over a set of p-nitrophenyl (*p*NP) esters of different lengths.

	Specific activity (U/mg protein)					
Ester	*p*NPC_2_	*p*NPC_3_	*p*NPC_4_	*p*NPC_8_	*p*NPC_10_	*p*NPC_12_
EstA_1_	76.43 ± 0.31	158.5 ± 2.5	204.8 ± 10.7	2.75 ± 0.14	0.62 ± 0.05	0.19 ± 0.09
EstA_2_	30.39 ± 0.19	50.56 ± 0.78	27.68 ± 0.83	1.60 ± 0.01	0.82 ± 0.08	0.16 ± 0.05
EstA_3_	30.40 ± 0.11	4.73 ± 0.05	1.16 ± 0.03	n.d.*^a^*	n.d.*^a^*	n.d.*^a^*
EstA_4_	28.39 ± 2.8	280.4 ± 8.7	196.1 ± 2.7	32.55 ± 2.67	11.19 ± 0.48	0.100 ± 0.03
EstA_5_	120.6 ± 5.3	665.9 ± 10.8	293.4 ± 14.1	8.11 ± 1.45	0.29 ± 0.02	0.17 ± 0.01
EstA_6_	520.1 ± 1.7	679.8 ± 9.8	467.7 ± 6.4	11.93 ± 0.06	0.37 ± 0.02	0.12 ± 0.01
EstA_7_	1.91 ± 0.03	2.18 ± 0.08	3.23 ± 0.06	0.53 ± 0.02	0.14 ± 0.01	0.02 ± 0.01
EstA_8_	14.07 ± 0.95	26.13 ± 0.71	70.39 ± 0.16	24.81 ± 0.09	0.95 ± 0.06	0.85 ± 0.02
EstB_1_	4.73 ± 0.33	15.18 ± 0.85	9.05 ± 0.61	4.25 ± 0.24	0.51 ± 0.04	0.24 ± 0.09
EstB_2_	2.32 ± 0.01	3.06 ± 0.03	2.17 ± 0.05	0.54 ± 0.04	0.12 ± 0.02	0.04 ± 0.01

*Values calculated from triplicates at pH 8.0 and 30°C.*

*^a^Not detected, activity level below detection limits under our assay conditions.*

*pNPC_2_, p-nitrophenyl acetate; pNPC_3_, propionate; pNPC_4_, butyrate; pNPC_8_, octanoate; pNPC_10_, decanoate; pNPC_12_, decanoate.*

**TABLE 3:** Specific activity (U/g pure protein) for each of the enzymes able to hydrolyze a set of structurally different plastic-related esters.

Substrate	Structure	Specific activity (U/g pure protein)							
		Est_*A*1_	Est_*A*2_	Est_*A*3_	Est_*A*4_	Est_*A*5_	Est_*A*6_	Est_*A*8_	Est_*B*2_
	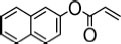								
2-Naphthyl acrylate		370.7 ± 20.3	37.7 ± 4.1	50.2 ± 0.4	1308 ± 79	n.d.*^a^*	n.d.*^a^*	748.1 ± 15.9	144.5 ± 10
	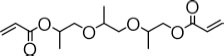								
Tri(propylene glycol) diacrylate		514.5 ± 36.3	4.9 ± 1.1	8.6 ± 0.7	3915 ± 48	n.d.*^a^*	n.d.*^a^*	1652 ± 75	73.5 ± 2.9
	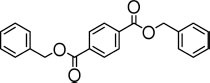								
Dibenzyl terephthalate		n.d.*^a^*	n.d.*^a^*	n.d.*^a^*	n.d.*^a^*	n.d.*^a^*	n.d.*^a^*	432.2 ± 27.5	n.d.*^a^*
	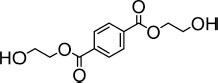								
BHET*^b^*		308.3 ± 3.9	5.0 ± 1.0	n.d.*^a^*	n.d.*^a^*	26.7 ± 1.7	12.3 ± 0.4	336.9 ± 3.6	91.1 ± 3.2

*Assays were performed in triplicate with values for each of the replicates given in the table with standard deviation. Values calculated at pH 8.0 and 30°C.*

*^a^Not detected, activity level below detection limits under our assay conditions.*

*^b^Time course of the degradation shown in Supplementary Figure 3.*

**FIGURE 2:**
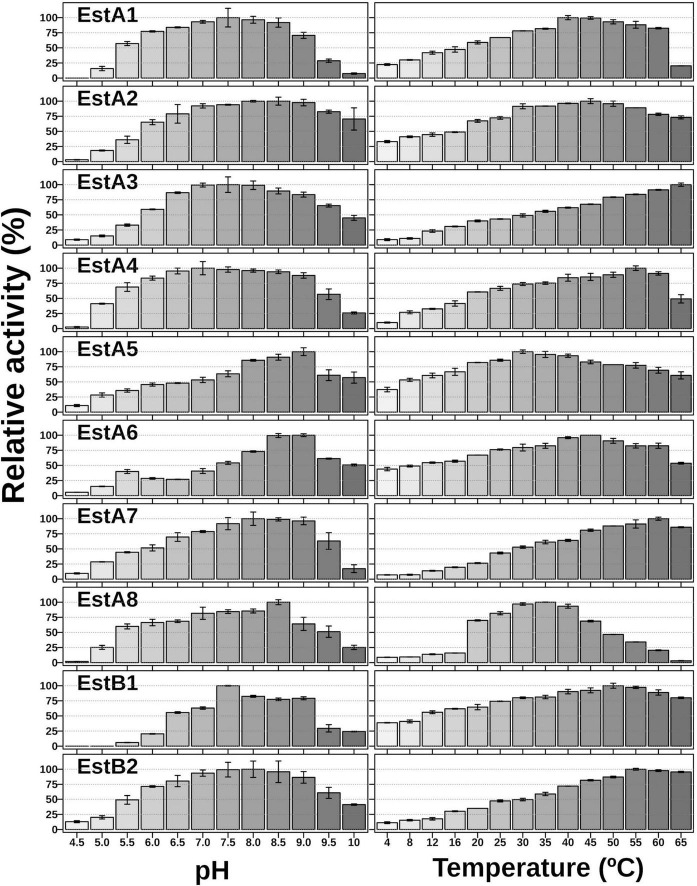
pH and thermal profiles of the purified enzymes. The data represent the relative percentages (%) of specific activity (U/g) in triplicates compared with the maximum activity using *p*NPC_3_ as substrate. For raw data see Supplementary Table 1.

Using *p*NPC_3_ as a substrate, the purified proteins were most active at temperatures ranging from 30 to 65°C (Figure 2). The average annual temperature in Los Rueldos is 13.8 ± 0.6°C, which varied from 10 ± 0.6°C to 17.1 ± 0.6 (Méndez-García et al., 2014), with a temperature of 17°C when samples were taken (July). At this value, the enzymes retained from 20 to 61% of the activity shown at the optimal temperature (Figure 2).

Using *p*NPC_3_ as a substrate, all enzymes showed an optimum pH for activity from neutral to slightly basic, which varied from 7.0 to 9.0 (Figure 2). This finding suggests that these proteins are most likely intracellularly produced, consistent with the absence of signal peptides in their sequences. Even though the enzymes showed a slightly basic optimum pH, all retained 33–68% of their activity at pH 5.5. Interestingly, Est_*A6*_ shows two activity peaks, one at pH 5.5 and one at pH 9.0, while Est_*A5*_, which only differs in two amino acids, has an optimum pH of 9.0 (Figure 2).

**FIGURE 3:**
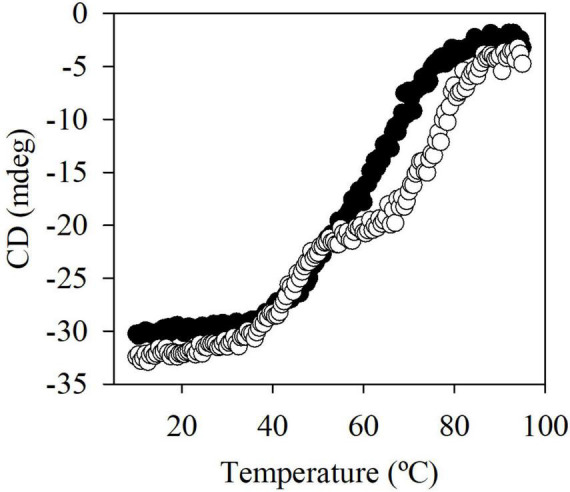
The thermal denaturation curve of Est_*A*5_ (filled circle) and Est_*A*6_ (open circle) at pH 7.0. The datasets were obtained by measuring the ellipticity changes at 220 nm obtained at different temperatures. For raw data see Supplementary Table 2.

**FIGURE 4:**
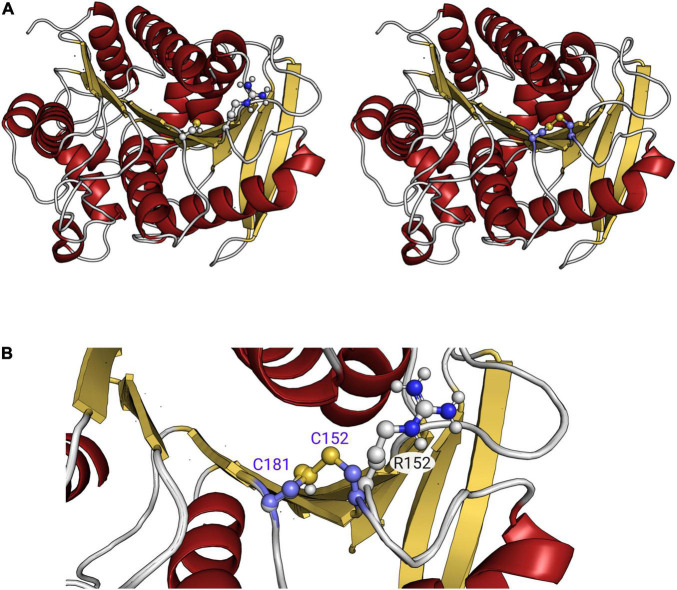
Three-dimensional comparison of the Est_*A*5_ and Est_*A*6_ hydrolases **(A)**. Zoom into the region that is different between both proteins, where the possible interaction of Cys152 and Cys181 in Est_*A*6_ can be seen in panel **(B)**. As shown, position 152 is occupied by Arg in Est_*A*5_ instead. Figure has been created using PMOL(TM) 2.2.3.

Sequence analysis revealed that EstA_5_ and EstA_6_ which have their origins in a bacterium of the phylum *Proteobacteria*, differ in only 2 amino acids (99.4% identity). Positions 152 and 172 are occupied by Arg and Ala in EstA_5_ and by Cys and Thr in EstA_6_, respectively. Notably, EstA_5_ was most active at 30°C, retaining more than 80% of the activity at temperatures from 20 to 45°C (Figure 2). The optimum temperature for activity increased up to 45°C for EstA_6_, which maintained more than 80% of its activity in the range from 30 to 60°C. Analysis by circular dichroism revealed that Est_*A*5_ showed a sigmoidal curve from which a temperature of denaturation of 60.4 ± 0.2°C could be obtained (Figure 3). However, the curve for Est_*A*6_ shows two transitions, one with a denaturation temperature of 48.1 ± 0.8°C and a second at 75.7 ± 0.2°C. The presence of these two phases may therefore indicate that the presence of these two amino acids may contribute to protein stability and its denaturation under distinct conditions. This result may explain the higher optimum temperature for the activity of this enzyme compared to Est_*A*5_, and the stabilization effect of Cys152 and Thr172. This difference in thermostability between Est_*A*5_ and Est_*A*6_ can probably be explained by the difference in amino acid 152, since Est_*A*6_ has a Cys that would allow it to make a possible disulfide bridge with Cys181, giving it greater thermostability than Est_*A*5_ (since Est_*A*5_ has an Arg at position 152 instead of a Cys), as shown by examination of the 3D models (Supplementary Figure 2 and Figure 4). It is plausible that this difference may also be responsible for the different pH profiles of both enzymes (Figure 2). If the disulfide bridge was present in Est_*A*6_, it could be removed by reduction or chemical modification. Activity tests revealed that both enzymes are resistant to the reducing agent beta-mercaptoethanol, with no activity lost even after 24 h of incubation at 30–45°C in the presence of a 1–10 mM inhibitor. By contrast, in the presence of the cysteine alkylating agent iodoacetamide, both enzymes were rapidly inactivated after 5 min. Thus, we could not verify the presumptive formation of the disulfide bridge in Est_*A*6_, and further studies are needed to test this assumption.

## Discussion

The effects of environmental constraints as prime forces shaping AMD populations have only begun to be elucidated through omics studies (Méndez-García et al., 2015). These effects are also of high interest in the context of the isolation and characterization of novel enzymes, for which limited data are available. However, the difficulty of cultivating organisms inhabiting AMD sites, which is due to their longer generation times, lower biomass yields, and cultivation conditions that are not yet fully understood, requires different strategies to overcome the problems associated not only with their cultivation, but also with the isolation of enzymes. Metagenomic approaches allow the screening of enzymes from such extreme environments. However, by using these tools, we have thus far explored only a small fraction of the enormous diversity on the planet, especially that of organisms inhabiting extremely acidic environments (Ferrer et al., 2015), again indicating the importance of establishing enzyme screening programs for AMD sites. The particular characteristics of the Los Rueldos AMD site (Méndez-García et al., 2015) that make it an interesting study site include the following. First, it is populated by a larger diversity of *Bacteria* and *Archaea* compared to other AMD sites, containing a total of 39 different species. Second, it has high microbial heterogeneity in local microniches defined by its O_2_ concentration gradients and spatial and biofilm architecture. As an example, only 1 of 18 species inhabiting the two distinct compartments in a stratified streamer investigated herein, namely, the oxic uppermost (B1A) and anoxic lowermost (B1B) sediment-attached strata, was shared. Therefore, it is plausible that Los Rueldos may also contain a greater diversity of microbial products such as enzymes.

We have sought to address this possibility by screening for esterases and lipases from the α/β-hydrolase fold superfamily in microbial communities inhabiting the Los Rueldos AMD site. These enzymes are desired tools for biocatalysis in a variety of industrial sectors (Daiha et al., 2015; Ferrer et al., 2015). Microorganisms that can survive under low pH values similar to those in Los Rueldos (pH ∼2) could be good sources of enzymes that can be used, for example, as additives in detergents, for the biobleaching of pulp and paper, in the clean-up of effluent streams from the textile processing industry, and in the degradation of plastics (Gomes et al., 2003; Adrio and Demain, 2014; Nchedo Ariole and George-West, 2020) and other polymers (Fütterer et al., 2004).

We used two complementary approaches for enzyme mining. A sequence-based metagenomic approach that searched for homologous enzymes in the metagenomic sequence data and function-driven screens in which expression libraries were used to identify, by using specific colorimetric substrates (Ferrer et al., 2016; Peña-García et al., 2016), clones containing enzymes of interest that could be missed in shallow metagenomics sequencing. By using both approaches, we identified 16 sequences that were potentially encoding esterases and lipases. The amino acid sequences were distantly related to sequences found in other AMD formations, which was in agreement with the distinct Los Rueldos-specific populations (Méndez-García et al., 2014). Indeed, we only observed some degree of sequence identity (27–54%) to 3 homologs from the Carnoulès (lead-zinc) mine, France (Bertin et al., 2011) in 6 of 16 sequences. In addition, the large differences among the recovered enzymes may correspond to the high population diversity that characterizes the Los Rueldos site (Méndez-García et al., 2014).

Notably, activity-based screens did not yield any active clones from the library created from the anoxic lowermost strata (B1B), while they yielded 10 active clones from the library created from the oxic uppermost strata (B1A). Thus, we searched for such enzymes in B1B by screening sequence data generated in a previous study (Méndez-García et al., 2014). It is plausible that the presence of low-O_2_-adapted microbial species in B1B, in contrast to the aerobic species in B1A, may account for the low efficiency of heterologous gene expression after cloning of the genetic material in the *E. coli* host and, possibly, the lower efficiency of the screening tests in the B1B library compared to that obtained for B1A. However, the fact that similar proportions of identified proteins (by naïve and sequence screening) in B1A (7 of 12) and B1B (2 of 4) could be produced as soluble active proteins when expressed in *E. coli* suggests that this may not be the only reason explaining the absence of positive clones in the B1B library. We cannot rule out that the native promoters of the partial genes from microorganisms inhabiting B1B cloned in the pCCFOS1 fosmids were inactive in *E. coli*, resulting in failed active clones on the plates. The data provided in Tables 2, 3 revealed that B1B enzymes were among the least-active enzymes among all hydrolases identified and characterized in the present study, and it is therefore also plausible that the low efficiency of the screening tests may have been due to the low activity level of enzymes from microorganisms inhabiting the anoxic B1B compartment. Additionally, it is plausible that different screening conditions (temperature, pH, inductor concentration, etc.) may be needed to detect other active proteins and that the enzymes from B1B would be more active under other assay conditions, the investigation of which is beyond the scope of the present study.

Regardless of the problems associated with the screening efficiency in different environments, including extreme AMD formations such as Los Rueldos, the analysis of the optimal pH profile of 10 out of the 16 hydrolases that could be produced in active form additionally revealed that their optimal pH was in the range from 7.0 to 9.0. This finding suggests that all hydrolases are presumptively produced intracellularly by acidophiles that thrive in the acidic Los Rueldos environment with a pH of 2.0. A similar phenotype has been found for other enzymes from AMD inhabitants, such as ATP-dependent DNA ligase from “*F. acidarmanus*” Fer1 (Jackson et al., 2007) and ene-reductase from “*Ferrovum*” sp. JA12 (Scholtissek et al., 2016), with pH optima of 6.0–7.0. However, we observed that most of the enzymes showed a slightly acid-stable phenotype, retaining ∼33–68% of activity at pH 5.5. It is plausible that the identification of enzymes with neutral-like pH optima is a consequence of screening tests performed at neutral pH, using a vector and host that allow mostly intracellular proteins to be produced and that presumably acid-stable enzymes could not be detected. In the future, performing naïve screens at such low pH values may help obtain additional active clones. However, while specific adaptations need to be explored in great detail, the retention of a high activity level at a slightly acidic pH might be attributed to the prevalence of acidic amino acids (negatively charged at a neutral pH) on the surfaces of these enzymes (Supplementary Figure 2), as reported for other proteins from acidophiles (Wu et al., 2020). Indeed, the relative frequencies of acidic residues in proteins in this study ranged from 67 to 54%, except for Est_*A*8_ (37%). As no major differences in pH profiles were observed when comparing the enzymes with the highest (Est_*A*1_: 67%) and lowest (Est_*A*8_: 37%) percentages of acidic residues, it is possible that other factors affect the activity and stability of the studied proteins from the Los Rueldos site. An example of this is the differences in stability against pH and temperature of Est_*A*5_ and Est_*A*6_, which show very different features and have only a two amino acid difference despite having the same percentage of acidic residues.

Furthermore, the biochemical properties of the esterases reported in this study revealed that all enzymes showed an activity–stability trade-off characteristic of mesophilic-adapted enzymes (from 30 to 65°C), which is a phenotype that has also been found for enzymes from other AMD inhabitants (Golyshina et al., 2006; Jackson et al., 2007; Ohara et al., 2014; Scholtissek et al., 2016). It is noticeable, however, that 5 of 10 characterized enzymes retained at least 50% of their maximal activity at temperatures as low as 12°C. The fact that the Los Rueldos site is characterized by a relatively low temperature compared to other AMD sites (Méndez-García et al., 2014) may account for this low-temperature-active phenotype. However, the lack of biochemical information on enzymes from other AMD sites does not allow us to validate this assumption.

Finally, it should be emphasized that the activity levels of the characterized enzymes (maximum for the best *p*NP substrates: approximately 680 to 3 U/mg, depending on the hydrolase) were in the range of other reported enzymes of different origins with esterase and lipase activities (Ferrer et al., 2015; Martínez-Martínez et al., 2018). The data suggest that the low-O_2_-adapted microbial species developed in the anoxic lowermost (B1B) sediment-attached strata do contain less-active enzymes than those developed in the oxic uppermost (B1A) strata under the conditions used herein. Whether this is typical *in vivo* or is a result of bias due to the assay conditions would require the characterization of a larger number of enzymes from both microenvironments in the Los Rueldos AMD system. The capacity of six of the enzymes from the Los Rueldos AMD formation, and thus the bacteria that contain them, for degrading acrylic- and terephthalate-like esters is noticeable. These enzymes could potentially be of use in developing plastic degradation strategies that have yet to be explored. In this context, the taxonomic distribution of top protein hits and the results of genome linguistics analysis suggested that the metagenomic fragments containing the six characterized enzymes that can potentially degrade plastic substrates most likely belong to *Actinobacteria* (genera related to *Acidithrix* and *Acidimicrobium*/*Ferrimicrobium*), *Acidobacteria*, and *Proteobacteria* (some are related to *Acidiphilium*). These are groups of acidophiles that have been largely neglected with respect to enzyme discovery.

Polyethylene terephthalate-degrading bioprospecting has shown that only a tiny fraction of carboxylic ester hydrolases can degrade PET and its intermediates BHET and MHET, including Actinomyces (i.e., *Thermobifida*, *Thermomonospora*, and *Saccharomonospora*), *Bacillus*, *Firmicutes* (e.g., *Clostridium*), *Bacteroidetes*, *Proteobacteria* (e.g., *Pseudomonas*, *Enterobacteria*, and *Ideonella sakaiensis*), and fungi [*Fusarium* and *Thermomyces* (*Humicola*)] (for a recent example, see Yoshida et al., 2016; Danso et al., 2018; Bollinger et al., 2020; Yan et al., 2021). In this study, we found that bacteria from the genera *Acidithrix* (the host of Est_*A*1_), *Acidimicrobium*/*Ferrimicrobium* (the host of Est_*A*2_), and unknown genera from the phylum *Proteobacteria* (the hosts of Est_*A*5_, Est_*A*6_, Est_*A*8_, and Est_*B*2_) could degrade BHET and could potentially degrade PET or PET oligomers under conditions yet to be explored, as no PET hydrolysis was detected under the assay conditions employed herein. Thus, the metagenomics approach applied herein expands the range of microorganisms containing enzymes supporting BHET hydrolysis and, possibly, PET depolymerization. For effective PET hydrolysis, in addition to a high degradation rate at 40–70°C, a broad range of pH stability (toward both the alkaline and acidic ranges) is one of the prerequisites of applied enzymes (Maurya et al., 2020), and these features were characteristic of some of the esterases from Los Rueldos reported herein. Further studies will reveal the catalytic efficiency and stability of hydrolases from Los Rueldos AMD systems to establish PET degradation systems or to support these systems in combination with other known PET-degrading enzymes.

It is plausible that the capacity to degrade plastic substrates comes from the adaptation of the active sites of enzymes to metabolize microbial polymeric substances that are naturally occurring in AMDs. Thus, in Los Rueldos, as in other AMDs such as the Richmond Mine at Iron Mountain (Jiao et al., 2010, 2011; Martínez-Martínez et al., 2013), the organisms present might contribute to the use/degradation of extracellular polymeric substances (EPS), which requires a broad range of enzymes (Flemming and Wingender, 2010).

## Data Availability Statement

The datasets presented in this study can be found in online repositories. The names of the repository/repositories and accession number(s) can be found in the article/Supplementary Material.

## Author Contributions

MF and AP conceived this study. MM-M contributed to gene cloning and expression. PV, MM-M, LF-L, and MF performed biochemical data and interpreted the data. SR and VG performed the 3D modeling. CM-G and MF contributed sample processing and library construction. OG contributed the phylogenetic analysis. MF drafted and revised the manuscript. All authors discussed, read, approved the manuscript, and authorized its submission for publication.

## Conflict of Interest

The authors declare that the research was conducted in the absence of any commercial or financial relationships that could be construed as a potential conflict of interest.

## Publisher’s Note

All claims expressed in this article are solely those of the authors and do not necessarily represent those of their affiliated organizations, or those of the publisher, the editors and the reviewers. Any product that may be evaluated in this article, or claim that may be made by its manufacturer, is not guaranteed or endorsed by the publisher.
