# Supplementary material for: Metagenomic Mining for Esterases in the Microbial Community of Los Rueldos Acid Mine Drainage Formation
Source: Front Microbiol. 2022 May 19;13:868839. doi: 10.3389/fmicb.2022.868839 (PMC9162777; doi:10.3389/fmicb.2022.868839)

Supplementary Material

**
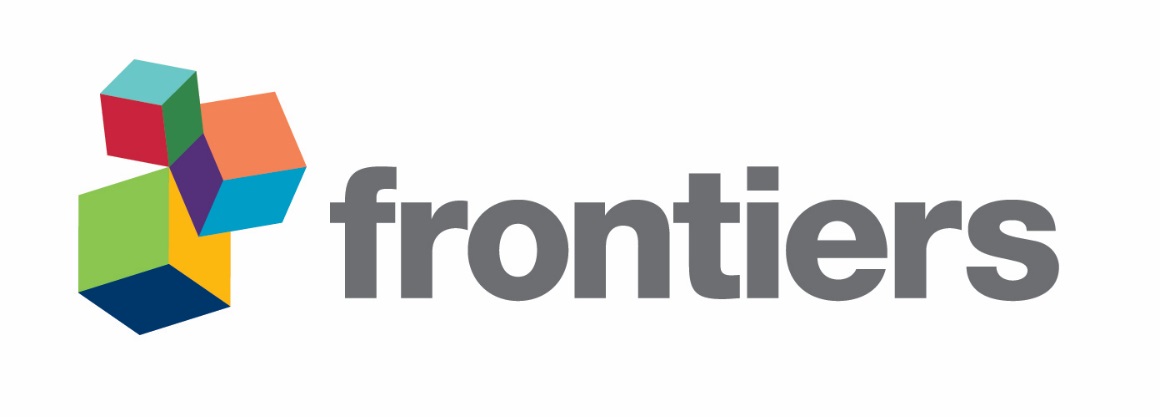
**

**Supplementary Methods.**

## Gene expression. Primers used for amplification are listed in Supplementary Methods. were as follows:

## EstA_1_ (Fw 5’-GAC GAC GAC AAG ATG CTCTCAAATGAAGCT-3’; Rv 5’-GA GGA GAA GCC CGG TCACCCAAGGATGAAC-3’)

## EstA_2_ (Fw 5’-GAC GAC GAC AAG ATG GAACCGATAGTAAATACCAC-3’; Rv 5’-GA GGA GAA GCC CGG TTA TCG CAA GCC TTT CC-3’)

## EstA_3_ (Fw 5’-GAC GAC GAC AAG ATG AAG ATT TTC TGT TGT C -3’; Rv 5’-GA GGA GAA GCC CGG T TA CTT CGT TCC CAG -3’)

## EstA_4_ (Fw 5’-GAC GAC GAC AAG ATG AAT AAA CTC GAT GAA TCG GCG-3’; Rv 5’-GA GGA GAA GCC CGG TTA TAA CGT CCG GAT GAA TG -3’)

## EstA_5_ (Fw 5’- GAC GAC GAC AAG ATG GCG ATG GAC CCT-3’; Rv 5’-GA GGA GAA GCC CGG T TA TGG TCC CCA AGC CT -3’)

## EstA_6_ (Fw 5’- GAC GAC GAC AAG ATG GCG ATG GAC CCT-3’; Rv 5’-GA GGA GAA GCC CGG T TA TGG TCC CCA AGC CT -3’)

## EstA_7_ (Fw 5’-GAC GAC GAC AAG ATG GAT ACC CAA CAA C -3’; Rv 5’-GA GGA GAA GCC CGG TCA GTC GCC CAT CTG-3’),

## EstA_8_ (Fw 5’-GAC GAC GAC AAG ATG CGT AAA CGG TTG ACA GTG G -3’; Rev 5’- GA GGA GAA GCC CGG T CA CCG GTG CGT CCG-3’)

## EstB_1_ (Fw 5’-GAC GAC GAC AAG ATG CCG GCG CAT TTT CCC -3’; Rv 5’-GA GGA GAA GCC CGG TCA CGT CAC CTC GCG CG -3’)

## EstB_2_ (Fw 5’-GAC GAC GAC AAG ATG GGC GTG AAA CCG -3’; Rv 5’-GA GGA GAA GCC CGG TCA ACT CGC CAG GCG -3’).

**Supplementary Table 1.** Raw dataset for pH and T optimal determination. Shown are the relative activity (R.a.) and standard deviation (STDV).

|  | **Est_A1_** | **Est_A1_** | **Es_tA2_** | **Est_A2_** | **Est_A3_** | **Est_A3_** | **Est_A4_** | **Est_A4_** | **Est_A5_** | **Est_A5_** |
| --- | --- | --- | --- | --- | --- | --- | --- | --- | --- | --- |
| **pH** | **R.a. (%)** | **STDV** | **R.a. (%)** | **STDV** | **R.a. (%)** | **STDV** | **R.a. (%)** | **STDV** | **R.a. (%)** | **STDV** |
| 4,50 | 0,00 | 0,00 | 3,04 | 0,42 | 8,95 | 1,00 | 2,66 | 0,69 | 10,86 | 1,76 |
| 5,00 | 15,74 | 3,60 | 18,33 | 0,85 | 15,16 | 1,50 | 41,20 | 0,82 | 28,28 | 3,49 |
| 5,50 | 57,12 | 3,34 | 36,08 | 5,99 | 33,07 | 1,82 | 68,80 | 7,20 | 35,71 | 2,71 |
| 6,00 | 77,28 | 0,99 | 65,20 | 4,17 | 59,13 | 0,65 | 83,52 | 3,35 | 45,61 | 2,61 |
| 6,50 | 83,90 | 0,87 | 78,99 | 15,39 | 86,71 | 1,27 | 95,28 | 4,82 | 48,02 | 0,66 |
| 7,00 | 93,11 | 2,23 | 92,17 | 3,55 | 99,35 | 3,26 | 100,00 | 10,94 | 53,27 | 4,43 |
| 7,50 | 100,00 | 15,45 | 94,07 | 0,76 | 100,00 | 12,86 | 97,71 | 4,38 | 63,38 | 4,90 |
| 8,00 | 96,44 | 5,68 | 99,91 | 1,23 | 98,99 | 7,05 | 95,98 | 2,57 | 85,77 | 1,46 |
| 8,50 | 91,85 | 7,71 | 100,00 | 6,62 | 89,53 | 4,87 | 94,02 | 2,97 | 90,88 | 4,89 |
| 9,00 | 70,66 | 5,04 | 97,63 | 5,39 | 83,54 | 4,09 | 87,92 | 4,52 | 100,00 | 6,46 |
| 9,50 | 28,64 | 2,81 | 82,57 | 2,63 | 65,34 | 2,43 | 56,83 | 8,72 | 61,07 | 8,94 |
| 10,00 | 7,49 | 1,16 | 70,50 | 18,37 | 45,05 | 4,12 | 25,90 | 1,60 | 57,13 | 9,18 |
| **Temp. (ºC)** | **R.a. (%)** | **STDV** | **R.a. (%)** | **STDV** | **R.a. (%)** | **STDV** | **R.a. (%)** | **STDV** | **R.a. (%)** | **STDV** |
| 4,00 | 22,33 | 1,44 | 33,03 | 1,79 | 9,00 | 1,66 | 9,97 | 0,95 | 37,32 | 3,78 |
| 8,00 | 30,03 | 0,66 | 41,05 | 1,42 | 11,02 | 1,23 | 26,99 | 2,38 | 53,28 | 2,67 |
| 12,00 | 41,96 | 2,51 | 44,61 | 2,82 | 23,23 | 2,16 | 32,62 | 0,95 | 60,65 | 3,90 |
| 16,00 | 47,32 | 4,46 | 48,86 | 0,79 | 30,85 | 0,70 | 41,56 | 4,39 | 66,68 | 5,86 |
| 20,00 | 58,96 | 2,59 | 67,36 | 2,30 | 40,01 | 1,55 | 60,64 | 0,34 | 82,06 | 0,30 |
| 25,00 | 67,01 | 0,03 | 72,35 | 2,30 | 43,12 | 0,45 | 66,61 | 3,27 | 85,88 | 1,75 |
| 30,00 | 78,12 | 0,26 | 91,46 | 4,14 | 49,07 | 2,58 | 73,75 | 2,43 | 100,00 | 2,84 |
| 35,00 | 81,70 | 0,93 | 91,83 | 0,31 | 55,90 | 1,71 | 75,29 | 1,98 | 95,37 | 4,99 |
| 40,00 | 100,00 | 3,42 | 96,38 | 0,55 | 61,96 | 0,86 | 84,21 | 5,78 | 93,29 | 2,71 |
| 45,00 | 99,49 | 2,16 | 100,00 | 4,07 | 67,61 | 0,45 | 85,51 | 5,84 | 82,96 | 2,99 |
| 50,00 | 93,10 | 3,48 | 95,78 | 4,35 | 79,31 | 0,53 | 89,05 | 4,34 | 78,55 | 0,00 |
| 55,00 | 88,19 | 5,69 | 89,01 | 0,08 | 83,94 | 0,60 | 100,00 | 3,78 | 77,37 | 4,62 |
| 60,00 | 82,58 | 1,19 | 78,19 | 1,97 | 100,00 | 0,73 | 91,30 | 2,80 | 69,31 | 4,90 |
| 65,00 | 20,30 | 0,17 | 73,08 | 2,48 | 91,41 | 2,74 | 49,23 | 6,88 | 60,73 | 5,90 |

**Supplementary Table 1.** Raw dataset for pH and T optimal determination. Shown are the relative activity (R.a.) and standard deviation (STDV).

|  | **Est_A6_** | **Est_A6_** | **Est_A7_** | **Est_A7_** | **Est_A8_** | **Est_A8_** | **Est_B1_** | **Est_B1_** | **Est_B2_** | **Est_B2_** |
| --- | --- | --- | --- | --- | --- | --- | --- | --- | --- | --- |
| **pH** | **R.a. (%)** | **STDV** | **R.a. (%)** | **STDV** | **R.a. (%)** | **STDV** | **R.a. (%)** | **STDV** | **R.a. (%)** | **STDV** |
| 4,50 | 5,52 | 0,16 | 9,52 | 0,97 | 1,98 | 0,12 | 5,99 | 0,00 | 12,89 | 1,68 |
| 5,00 | 15,24 | 0,50 | 28,57 | 0,32 | 25,26 | 3,32 | 20,37 | 0,00 | 20,07 | 2,73 |
| 5,50 | 39,85 | 3,26 | 44,50 | 0,97 | 59,93 | 4,14 | 55,65 | 0,31 | 48,97 | 7,18 |
| 6,00 | 28,53 | 1,34 | 51,64 | 5,08 | 66,36 | 5,34 | 63,05 | 0,49 | 71,50 | 1,32 |
| 6,50 | 26,97 | 0,32 | 69,68 | 7,18 | 68,54 | 2,19 | 78.16 | 1,38 | 80,46 | 9,29 |
| 7,00 | 40,68 | 4,10 | 78,73 | 1,57 | 81,72 | 10,08 | 83,05 | 1,93 | 93,55 | 5,16 |
| 7,50 | 54,18 | 2,45 | 91,80 | 10,05 | 84,47 | 3,04 | 100,00 | 0,51 | 99,32 | 12,11 |
| 8,00 | 72,98 | 1,20 | 100,00 | 11,06 | 85,51 | 3,29 | 82,43 | 1,59 | 100,00 | 13,55 |
| 8,50 | 99,40 | 3,32 | 98,89 | 2,69 | 100,00 | 4,13 | 77,51 | 2,16 | 95,76 | 17,87 |
| 9,00 | 100,00 | 2,38 | 96,35 | 6,29 | 64,12 | 10,87 | 79,22 | 2,43 | 86,51 | 9,60 |
| 9,50 | 61,26 | 0,80 | 63,17 | 13,72 | 51,23 | 9,38 | 69,53 | 6,06 | 60,73 | 9,30 |
| 10,00 | 50,77 | 1,49 | 17,30 | 6,45 | 25,20 | 3,57 | 24,26 | 0,49 | 41,16 | 1,19 |
| **Temp. (ºC)** | **R.a. (%)** | **STDV** | **R.a. (%)** | **STDV** | **R.a. (%)** | **STDV** | **R.a. (%)** | **STDV** | **R.a. (%)** | **STDV** |
| 4,00 | 43,94 | 2,87 | 6,99 | 0,38 | 8,85 | 0,32 | 38,72 | 0,42 | 11,21 | 1,50 |
| 8,00 | 48,95 | 1,54 | 7,24 | 0,91 | 9,44 | 0,14 | 40,95 | 2,41 | 15,35 | 1,15 |
| 12,00 | 54,49 | 1,09 | 13,73 | 0,72 | 13,85 | 0,81 | 56,10 | 2,31 | 17,64 | 2,08 |
| 16,00 | 56,99 | 1,72 | 19,66 | 0,72 | 15,94 | 0,19 | 61,86 | 0,65 | 30,19 | 0,69 |
| 20,00 | 67,06 | 0,14 | 26,45 | 0,91 | 69,90 | 1,05 | 64,63 | 4,44 | 34,93 | 0,06 |
| 25,00 | 76,10 | 1,04 | 43,26 | 1,46 | 81,76 | 2,60 | 74,29 | 0,47 | 47,35 | 1,74 |
| 30,00 | 79,55 | 5,66 | 52,98 | 1,94 | 97,04 | 2,05 | 80,12 | 1,38 | 49,54 | 2,09 |
| 35,00 | 82,44 | 4,11 | 61,36 | 2,96 | 100,00 | 0,27 | 81,30 | 2,83 | 58,74 | 2,73 |
| 40,00 | 95,87 | 1,56 | 64,13 | 1,77 | 93,45 | 3,34 | 90,29 | 3,78 | 72,12 | 0,20 |
| 45,00 | 100,00 | 0,00 | 81,02 | 1,91 | 68,74 | 1,32 | 92,34 | 4,06 | 81,81 | 1,06 |
| 50,00 | 90,61 | 4,00 | 88,00 | 0,14 | 46,67 | 0,20 | 100,00 | 4,17 | 87,13 | 1,40 |
| 55,00 | 82,39 | 3,69 | 91,27 | 6,79 | 34,24 | 0,20 | 97,34 | 1,94 | 100,00 | 1,47 |
| 60,00 | 82,53 | 4,29 | 100,00 | 2,55 | 20,49 | 0,74 | 88,80 | 4,37 | 97,79 | 1,45 |
| 65,00 | 53,65 | 1,43 | 85,92 | 0,78 | 3,18 | 0,24 | 80,06 | 1,24 | 95,57 | 1,06 |

**Supplementary Table 2.** Raw CD dataset. Shown are are the ellipticity (mdeg) at increasing temperature.

|  | Est_A5_ | Est_A6_ |
| --- | --- | --- |
| Temperature [ºC] | CD[mdeg] | CD[mdeg] |
| 10 | -30,2355 | -32,4134 |
| 10,5 | -30,4505 | -32,1925 |
| 11 | -30,1818 | -32,7964 |
| 11,5 | -30,2753 | -32,5341 |
| 12 | -29,9322 | -32,1362 |
| 12,5 | -30,1264 | -32,8926 |
| 13 | -30,1046 | -32,2501 |
| 13,5 | -30,3353 | -31,9953 |
| 14 | -30,1666 | -32,3313 |
| 14,5 | -30,1063 | -32,133 |
| 15 | -30,023 | -31,5756 |
| 15,5 | -30,2941 | -32,0506 |
| 16 | -29,7303 | -31,8828 |
| 16,5 | -30,4241 | -31,5579 |
| 17 | -29,6339 | -32,04 |
| 17,5 | -30,0828 | -32,3753 |
| 18 | -29,8326 | -31,9734 |
| 18,5 | -29,508 | -32,0717 |
| 19 | -29,4282 | -32,3922 |
| 19,5 | -29,9124 | -32,1287 |
| 20 | -29,6403 | -32,0998 |
| 20,5 | -30,1774 | -32,1637 |
| 21 | -29,6405 | -31,9757 |
| 21,5 | -29,571 | -31,8858 |
| 22 | -29,6513 | -31,7362 |
| 22,5 | -29,8618 | -31,8179 |
| 23 | -29,7841 | -32,1924 |
| 23,5 | -30,0507 | -31,9507 |
| 24 | -29,6078 | -31,1929 |
| 24,5 | -29,4857 | -32,12 |
| 25 | -29,8984 | -31,4859 |
| 25,5 | -29,7775 | -31,546 |
| 26 | -29,8453 | -31,1239 |
| 26,5 | -29,8418 | -31,2003 |
| 27 | -29,3093 | -31,1083 |
| 27,5 | -29,669 | -31,2172 |
| 28 | -29,9344 | -31,5244 |
| 28,5 | -29,5828 | -31,43 |
| 29 | -29,231 | -31,1209 |
| 29,5 | -29,3039 | -30,8467 |
| 30 | -29,5449 | -31,46 |
| 30,5 | -29,6149 | -31,099 |
| 31 | -29,4589 | -30,9628 |
| 31,5 | -29,4492 | -30,5563 |
| 32 | -29,1089 | -31,1811 |
| 32,5 | -29,3244 | -31,4255 |
| 33 | -29,6845 | -30,5719 |
| 33,5 | -29,4601 | -30,466 |
| 34 | -29,3836 | -30,5156 |
| 34,5 | -29,1172 | -29,9337 |
| 35 | -29,6838 | -30,2223 |
| 35,5 | -28,9097 | -30,2021 |
| 36 | -29,6783 | -30,6921 |
| 36,5 | -29,2074 | -30,1014 |
| 37 | -28,9754 | -29,6699 |
| 37,5 | -28,7465 | -29,255 |
| 38 | -28,558 | -29,3116 |
| 38,5 | -28,2054 | -28,6664 |
| 39 | -28,7227 | -28,75 |
| 39,5 | -28,6049 | -28,2467 |
| 40 | -28,2276 | -28,2796 |
| 40,5 | -27,4889 | -28,5577 |
| 41 | -27,1775 | -28,5072 |
| 41,5 | -27,6651 | -28,1992 |
| 42 | -27,0429 | -27,4648 |
| 42,5 | -26,7985 | -27,1775 |
| 43 | -26,5033 | -26,6866 |
| 43,5 | -27,0613 | -25,5988 |
| 44 | -26,9869 | -25,8114 |
| 44,5 | -26,5802 | -26,2712 |
| 45 | -26,3899 | -25,5385 |
| 45,5 | -26,0552 | -24,5236 |
| 46 | -26,4289 | -25,0246 |
| 46,5 | -25,3416 | -24,3748 |
| 47 | -25,3504 | -23,8745 |
| 47,5 | -25,4345 | -23,4871 |
| 48 | -24,955 | -23,4763 |
| 48,5 | -23,9 | -22,4559 |
| 49 | -23,7595 | -23,0857 |
| 49,5 | -23,5134 | -22,7893 |
| 50 | -22,6119 | -22,6223 |
| 50,5 | -22,4925 | -21,995 |
| 51 | -22,748 | -22,032 |
| 51,5 | -21,8602 | -21,6372 |
| 52 | -21,2436 | -21,5761 |
| 52,5 | -21,591 | -21,1701 |
| 53 | -20,7933 | -21,5989 |
| 53,5 | -20,9151 | -21,4986 |
| 54 | -20,6554 | -21,7953 |
| 54,5 | -19,5563 | -21,7895 |
| 55 | -20,3365 | -20,2711 |
| 55,5 | -20,3925 | -20,7506 |
| 56 | -19,1723 | -21,0985 |
| 56,5 | -18,9248 | -20,6003 |
| 57 | -18,5267 | -21,3938 |
| 57,5 | -17,5441 | -21,4605 |
| 58 | -18,0785 | -20,619 |
| 58,5 | -17,7834 | -20,1407 |
| 59 | -16,6941 | -20,2856 |
| 59,5 | -17,2088 | -19,9886 |
| 60 | -17,8139 | -20,6537 |
| 60,5 | -16,1287 | -20,7549 |
| 61 | -16,0604 | -20,4703 |
| 61,5 | -14,8908 | -19,5051 |
| 62 | -15,2469 | -20,4376 |
| 62,5 | -13,8934 | -20,0305 |
| 63 | -14,5652 | -20,2402 |
| 63,5 | -13,5921 | -19,3495 |
| 64 | -13,8487 | -19,8472 |
| 64,5 | -12,4038 | -19,5439 |
| 65 | -12,2446 | -19,1735 |
| 65,5 | -12,2543 | -18,029 |
| 66 | -12,7342 | -19,9237 |
| 66,5 | -11,2134 | -18,5189 |
| 67 | -10,669 | -19,7819 |
| 67,5 | -11,1888 | -17,5021 |
| 68 | -10,3364 | -18,2953 |
| 68,5 | -9,38336 | -17,3366 |
| 69 | -7,5344 | -18,249 |
| 69,5 | -9,54896 | -17,7095 |
| 70 | -7,29724 | -16,7999 |
| 70,5 | -7,98115 | -16,1397 |
| 71 | -9,19779 | -16,1932 |
| 71,5 | -7,27887 | -15,1218 |
| 72 | -7,02566 | -14,8354 |
| 72,5 | -6,92346 | -13,992 |
| 73 | -6,05708 | -13,9309 |
| 73,5 | -5,93789 | -14,95 |
| 74 | -6,66295 | -15,0232 |
| 74,5 | -6,38058 | -13,7499 |
| 75 | -5,12029 | -13,1926 |
| 75,5 | -4,80027 | -13,3898 |
| 76 | -4,60327 | -12,0202 |
| 76,5 | -4,81468 | -11,2485 |
| 77 | -4,14251 | -12,137 |
| 77,5 | -4,03997 | -10,0443 |
| 78 | -4,74186 | -9,33663 |
| 78,5 | -4,43912 | -10,2581 |
| 79 | -4,33824 | -8,79568 |
| 79,5 | -3,25897 | -7,37057 |
| 80 | -3,92889 | -6,80189 |
| 80,5 | -3,40065 | -7,90646 |
| 81 | -3,35938 | -7,46108 |
| 81,5 | -3,71217 | -7,29796 |
| 82 | -3,53548 | -5,39368 |
| 82,5 | -3,53273 | -7,07071 |
| 83 | -3,16077 | -6,43723 |
| 83,5 | -3,15472 | -5,90897 |
| 84 | -3,25947 | -5,5136 |
| 84,5 | -2,23799 | -5,2936 |
| 85 | -3,3684 | -5,88405 |
| 85,5 | -2,73738 | -5,19587 |
| 86 | -2,66419 | -4,6673 |
| 86,5 | -2,49089 | -3,80432 |
| 87 | -2,5217 | -4,28439 |
| 87,5 | -2,97482 | -4,10094 |
| 88 | -1,86086 | -3,83238 |
| 88,5 | -2,37473 | -4,00178 |
| 89 | -2,49967 | -4,31567 |
| 89,5 | -2,22309 | -4,22339 |
| 90 | -2,49247 | -3,98982 |
| 90,5 | -2,52036 | -5,44624 |
| 91 | -2,50391 | -3,61323 |
| 91,5 | -2,82115 | -4,21508 |
| 92 | -1,83269 | -3,95576 |
| 92,5 | -2,0883 | -3,5294 |
| 93 | -1,83587 | -3,51918 |
| 93,5 | -2,34054 | -4,37046 |
| 94 | -3,11969 | -3,22942 |
| 94,5 | -2,44003 | -3,81639 |
| 95 | -3,20239 | -4,75636 |

**Supplementary Figure 1.** SDS-PAGE gel of purified esterases from the Los Rueldos AMD system.

**
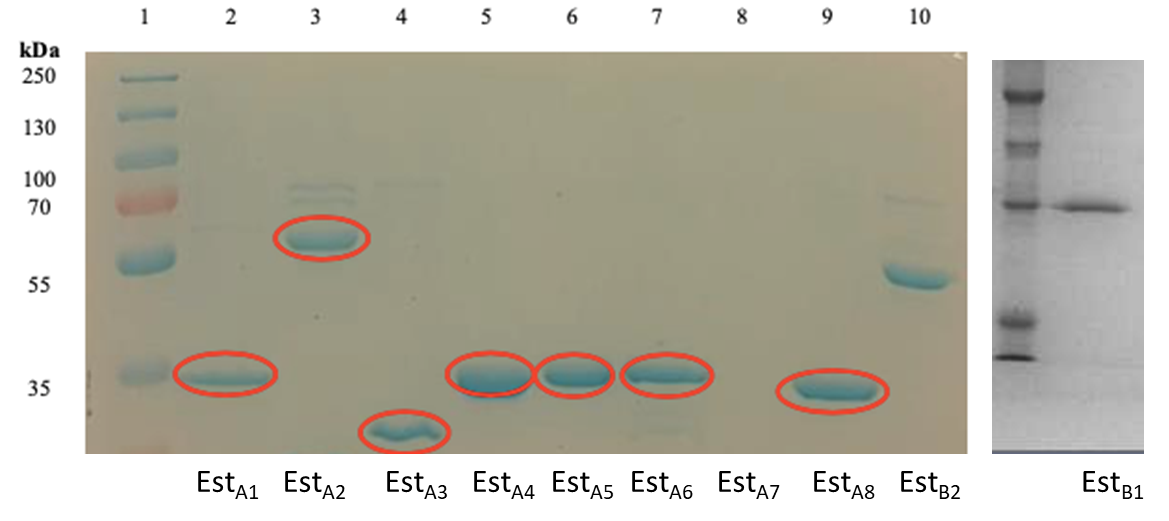
**

**Supplementary Figure 2 ǀ** Distribution of charged residues on the protein's surface of esterases from Los Rueldos AMD system. The surfaces are displayed in white, the acid residues in red, and the basic residues in blue. The surface representation is shown in the 2 sides of the protein structure.


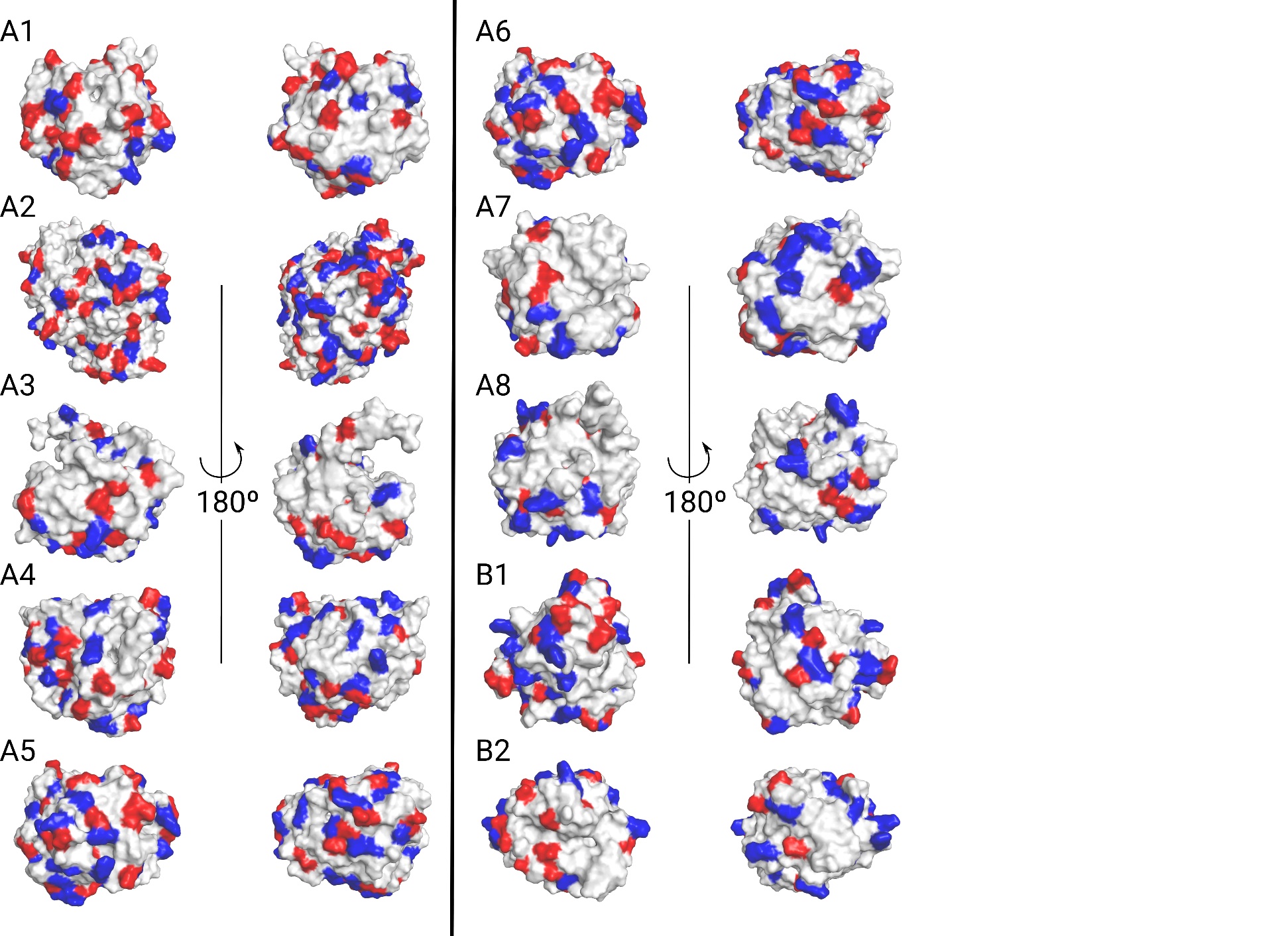


**Supplementary Figure 3 ǀ** Representative time course curve for the hydrolysis of BHET by Est_A1_, Est_A2_, Est_A5_, Est_A6_, and Est_A8_ and Est_B2_. Reactions conditions: [protein]: 270 μg/ml; [BHET]: 20 mM; reaction volume: 44 μl; T: 30 °C; and pH: 8.0. Hydrolysis was followed spectrophotometrically in continuous mode at 550 nm. The time course for a control reaction (no enzyme added) is also shown.


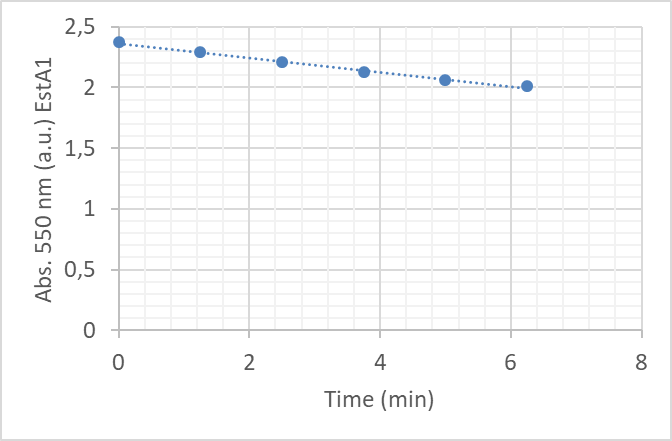

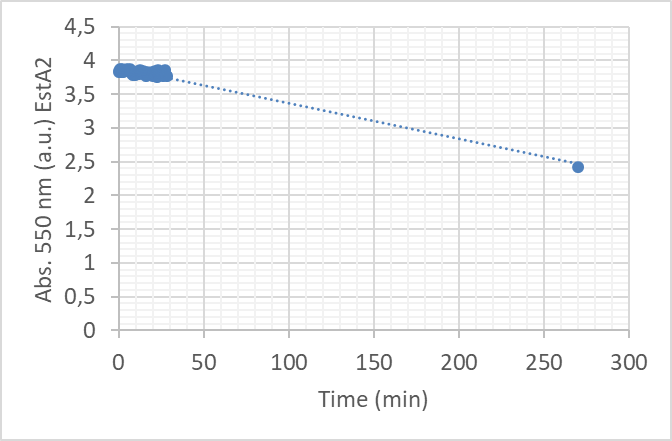

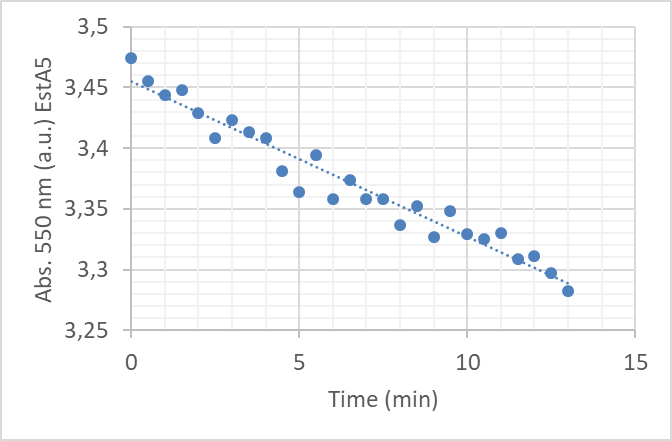


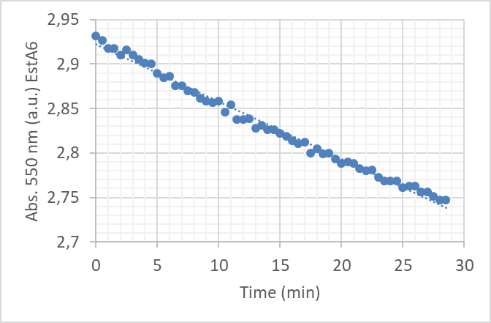

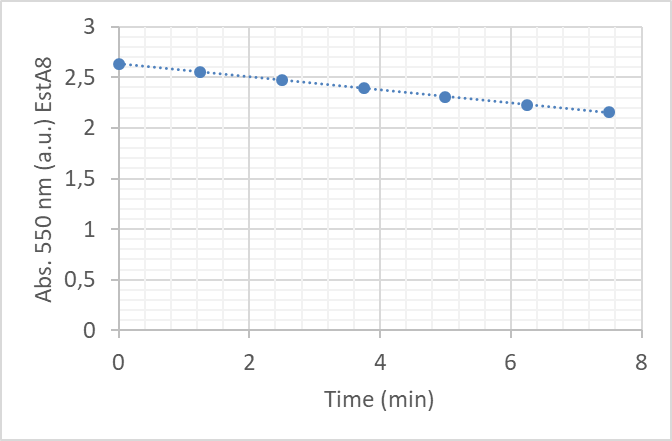

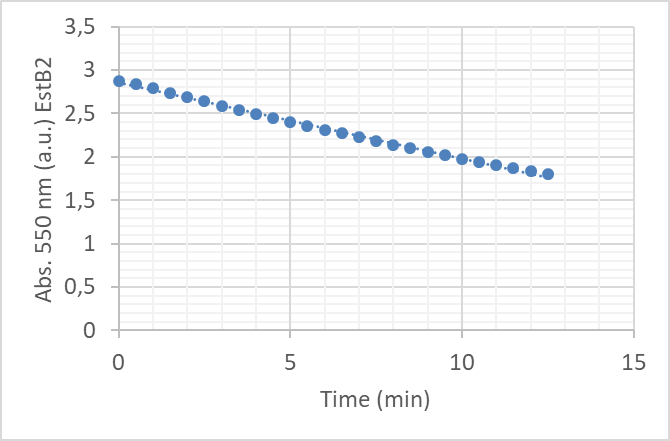


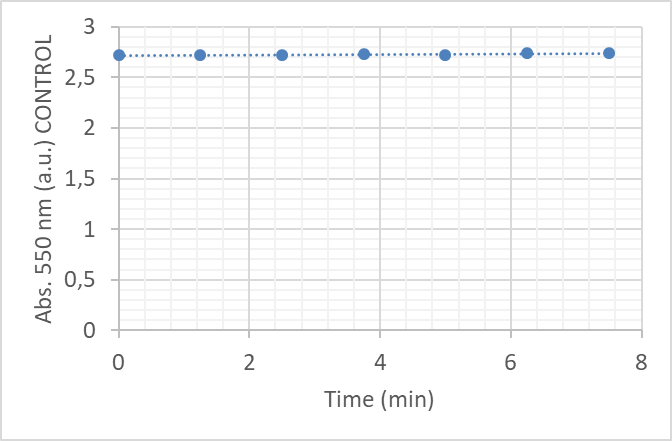

Supplement: Supplementary file 1 [file Data_Sheet_1.docx]
